# Post-Process Cytotoxicity of Resins in Clear Aligner Fabrication

**DOI:** 10.3390/polym17131776

**Published:** 2025-06-26

**Authors:** Sabahattin Bor, Yeşim Kaya, Ayşe Demiral, Mustafa Güngörmüş

**Affiliations:** 1 Department of Orthodontics, Faculty of Dentistry, İnönü University, Malatya 44280, Türkiye; 2 Department of Orthodontics, Faculty of Dentistry, Ankara Yıldırım Beyazıt University, Ankara 06760, Türkiye; ykaya@aybu.edu.tr; 3 Department of Translational Medicine, Institute of Health Sciences, Ankara Yıldırım Beyazıt University, Ankara 06760, Türkiye; 215333401@aybu.edu.tr; 4 Department of Basic Medical Sciences, Faculty of Dentistry, Ankara Yıldırım Beyazıt University, Ankara 06760, Türkiye; mgungormus@aybu.edu.tr

**Keywords:** 3D-printed clear aligners, Tera Harz TC-85 DAC resin, Clear-A resin, Cytotoxicity

## Abstract

This study aimed to evaluate the cytotoxicity of two resin materials, Tera Harz TC-85 DAC and Clear-A, along with the effects of two different post-printing protocols applied to Clear-A. Samples were produced using the Ackuretta Sol printer. The following three groups were formed based on the resins used and the post-curing methods applied: Group 1: Tera Harz TC-85 DAC resin + Tera Harz Cure; Group 2: Clear-A resin + Curie machine; and Group 3: Clear-A resin + Tera Harz Cure. All samples were sterilized in 70% ethanol for 5 min, rinsed with sterile deionized water, and incubated in Dulbecco’s Modified Eagle Medium at 37 °C for 72 h. Cytotoxicity assessment was performed by the XTT and RTCA methods using the human gingival fibroblast cell line. According to the XTT assay, undiluted resin extracts exhibited approximately 75–80% cell viability at 24 h, while further dilutions resulted in a viability exceeding 90%. No significant differences in viability were observed among the groups at any dilution at 48 and 72 h. The xCELLigence RTCA results aligned with the XTT findings, showing a transient decrease in cell viability within the first 24 h, followed by continued cell growth. This study demonstrated that extracts from all tested 3D-printed resins exhibited biocompatibility with human gingival fibroblasts. These findings support their potential for further applications in the dental and biomedical fields.

## 1. Introduction

Since the founding of Align Technology in 1998 and the subsequent entry of other companies into the market, clear aligner treatments have gained increasing popularity among both patients and clinicians [1]. Initially, clear aligners were fabricated using an indirect process known as thermoforming, in which 3D-printed study models were produced, followed by the application of pressure and heat to mold the aligner material over these models [2]. The involvement of aligner companies and laboratories in the manufacturing process introduced additional costs and delays in aligner delivery [3,4]. To address these challenges, clinicians began fabricating in-house clear aligners with the development and widespread adoption of resin-based 3D printers [5]. However, the combined processes of the serial 3D printing of study models and thermoforming remain both time-intensive and costly, while also raising concerns regarding the environmental impact of plastic waste [6,7].

Recent advancements in 3D printing technologies have enabled the direct fabrication of clear aligners, offering several advantages [8,9]. The custom design capabilities of 3D-printed clear aligners allow for precise adjustments in aligner thickness, enabling specific areas to be made thicker or thinner as needed to optimize force application in the intended direction [9]. Additionally, the direct 3D printing of clear aligners minimizes the geometric and dimensional distortions associated with the thermoforming process, thereby increasing accuracy and efficiency [8,10]. The ability to both plan and manufacture 3D-printed clear aligners in-house further enhances their time and cost efficiency [4,7].

Directly 3D-printed clear aligners are fabricated through the photopolymerization of a photosensitive liquid resin. While these resin materials are highly toxic before polymerization, their toxicity decreases significantly following post-polymerization procedures, which involve the removal of uncured resin [6,7]. After 3D printing, clear aligners are subjected to a post-curing process using ultraviolet (UV) light, which serves to complete the polymerization of the resin and improve its biocompatibility [7,11]. The post-curing process is performed in accordance with the manufacturer’s recommendations to reduce toxicity and increase hardness [11,12]. It is well established that UV-initiated free radical polymerization is sensitive to oxygen, which inhibits the completion of photopolymerization [7,12,13]. To mitigate this issue, manufacturers have developed post-curing devices that utilize inert gases like helium, carbon dioxide, and nitrogen to generate oxygen-free curing environments [12,13].

The market currently offers only a limited selection of resins specifically designed for the direct production of clear aligners. Among these, Tera Harz TC 85 DAC (Graphy, Seoul, Republic of Korea) was the first resin developed for this purpose and has received approval from both the U.S. Food and Drug Administration (FDA) and Conformité Européenne (CE). Clear A (Senertek, İzmir, Turkey) is another available resin, which was initially certified only by CE. However, following the completion of this study, the updated version, Clear A Version 2, also received FDA approval. Unlike other manufacturers, the producer of Clear-A does not require the use of professional 3D printers or post-curing units equipped with nitrogen gas. Instead, they recommend a post-curing process using a glass container filled with glycerol.

A comprehensive review of the literature revealed that only a limited number of studies have investigated the cytotoxicity of 3D-printed clear aligner resins [4,7,14,15]. Among these studies, variations were observed in terms of post-curing devices [7], curing durations within the same device [15], cleaning protocols [14], and resin type [4], all of which focused specifically on the Tera Harz TC-85 DAC resin. However, to date, no study has compared the cytotoxicity of different 3D-printed clear aligner resins. In light of this gap, the primary aim of the present study was to compare the cytotoxicity of the Tera Harz TC-85 DAC and Clear-A resins. The secondary aim was to evaluate the effect of two different post-curing protocols on the cytotoxicity of the Clear-A resin. TC-85 DAC and Clear-A were selected due to their commercial availability, CE certification, and specific marketing for direct 3D-printed aligner fabrication. TC-85 DAC has also received FDA and KFDA approval, further supporting its clinical applicability. The respective curing devices, Graphy THC2 and Curie Cure, were chosen in line with manufacturer recommendations to ensure clinically relevant processing. The alternative hypothesis (H_1_) was that the cytotoxicity of directly 3D-printed clear aligners may vary depending on the resin material and post-curing process used.

## 2. Materials and Methods

### 2.1. Preparation of Clear Aligner Samples

**Figure 1:**
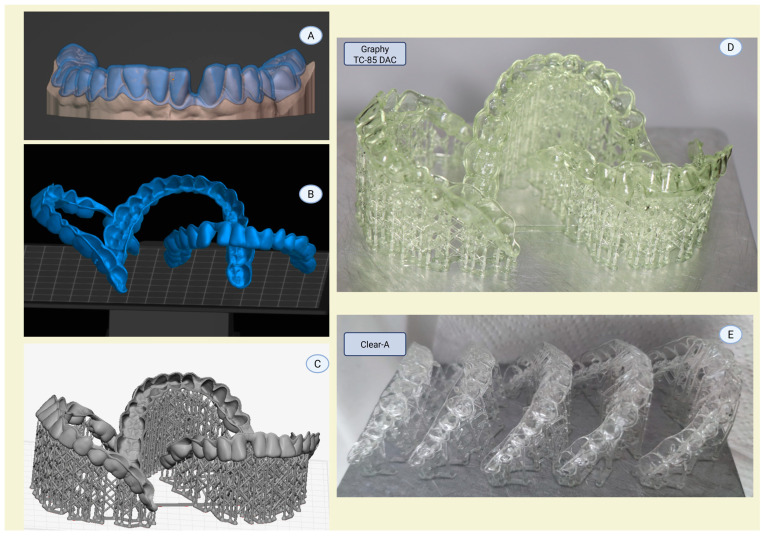
From Digital Design to Post-Printing: Fabrication of Shape-Memory Aligners with Resin-Based Polymers. (**A**) Aligner design; (**B**) orientation on the building platform; (**C**) addition of support structures in a 3D printing slicer program; (**D**) post-printed Graphy TC-85 DAC aligner (greenish color); and (**E**) post-printed Clear-A aligner (transparent).

Rectangular aligner samples (10 mm × 12 mm; thickness: 0.6 mm) produced from two brands of clear aligner resin (Tera Harz TC-85 DAC (Graphy, Seoul, Republic of Korea) and Clear-A (Senertek, İzmir, Turkey)) were used for direct aligner fabrication in this in vitro study. The samples were designed in Blenderfordental software (Blenderfordental 2024, Dubai, United Arab Emirates) with a thickness of 0.6 mm. An Ackuretta SOL printer (Ackuretta, Taipei, Taiwan) was used to 3D print these samples with a layer thickness of 100 µ and a 45° print angulation. Figure 1 shows the immediate post-printing appearance of the aligners prior to post-curing. The Graphy TC-85 DAC aligner (D) exhibited a greenish color, while the Clear-A aligner (E) appeared transparent. These color differences disappeared following the post-curing process, with both aligners becoming uniformly clear.

Three experimental groups were established. In the first group, Tera Harz TC-85 DAC resin was used following the manufacturer’s instructions. Upon completion of the 3D printing process, the samples were removed from the printing platform and subjected to a 6-min cleaning cycle in a centrifuge machine with water to remove uncured residual resin. Then, the supports were removed and dried with compressed air.

The post-curing process was performed for 25 minutes using a nitrogen generator curing machine, Tera Harz Cure (THC2, Graphy, Seoul, Republic of Korea). Following the post-curing process, the samples were subjected to ultrasonic cleaning in warm water for 1 min, followed by thermal treatment in boiling water at 100 °C for 1 min.

In the second group, Clear-A resin was used in accordance with the manufacturer’s instructions. Following the 3D printing process, the samples were removed from the printing platform, cleaned in a centrifuge machine with water for 6 min, and then had their supports removed before being dried with compressed air. The post-curing process was conducted in a glass container filled with glycerin for 10 min, using a Curie Cure device (Ackuretta, Taipei City, Taiwan). Afterwards, the samples were subjected to ultrasonic cleaning in warm water for 1 min, followed by thermal treatment in boiling water at 100 °C for 1 min.

**Figure 2:**
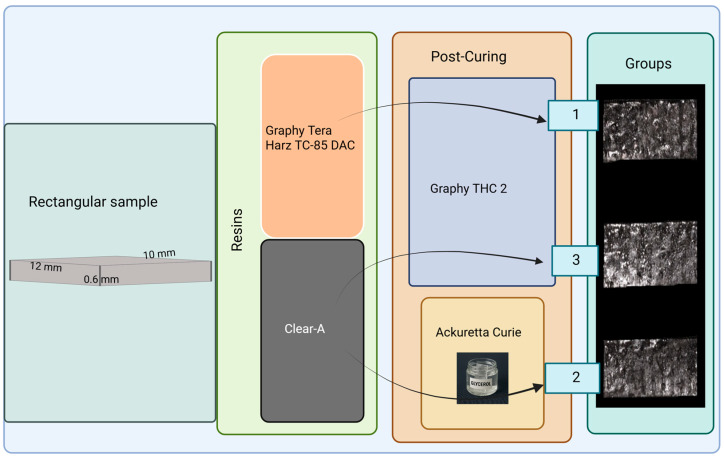
Production of rectangular samples by groups using two different resins and post-curing devices. **Group 1**: Tera Harz TC-85 DAC resin, post-cured using the Graphy THC 2. **Group 2**: Clear-A resin, post-cured in a glycerin-filled glass container using the Ackuretta Curie device. **Group 3**: Clear-A resin, post-cured using Graphy’s THC 2. Created with BioRender.com.

In the third group, Clear-A resin was used with a post-curing procedure identical to that of the first group. Hence, as illustrated in Figure 2, three distinct groups were established. All 3D printing and post-curing procedures were performed by the same operator (S.B.).

### 2.2. Preparation of the Extracts

The extracts of the 3D-printed samples for each group were prepared in accordance with the ISO 10993-12 Biological evaluation of medical devices—Sample preparation and reference materials standard [16].

Briefly, the 3D-printed samples were disinfected in 70% ethanol solution for 5 min and rinsed with sterile de-ionized water. This method was selected due to the lack of access to an ethylene oxide (EO) gas sterilizer and in order to avoid introducing additional cytotoxic by-products known to result from EO sterilization. UV sterilization was also avoided to prevent possible further polymerization or degradation of the UV-cured materials. Then, the 3D-printed samples were immersed in Dulbecco’s Modified Eagle Medium (DMEM) containing 10% Fetal Bovine Serum (FBS) and 1% Penicillin–streptomycin (PS). The surface area of each rectangular 3D-printed sample was calculated using a caliper, and a liquid volume of 6 cm^2^/mL, which corresponded to two whole specimens and 1st/3rd of a specimen, was used. The positive control group extracts were prepared using natural rubber latex, following the same extraction procedures and conditions as applied to the other test groups. Annex B of ISO 10993-5 lists natural rubber latex among the materials that can be used effectively as positive controls in cytotoxicity assays [17]. The 3D-printed samples were kept in the DMEM at 37 °C for 72 h, and the resulting extracts were used immediately upon preparation. The human gingival fibroblast (HGF) cell line (PCS-201-018™) was used for the cytotoxicity assessments.

### 2.3. XTT Cell Viability Assay

HGF cells (passage number 5) stored in liquid nitrogen were rapidly thawed in a 37 °C water bath. The cells were counted with a TC20 automated cell counter (BioRad, Hercules, CA, USA) and 5 × 10^3^ cells were seeded to a 96-well plate. Separate sets of cells were prepared for 24 h, 48 h, and 72 h measurements. The cells were grown for 24 h at 37 °C in a 5% CO_2_ atmosphere in DMEM containing 10% FBS and 1% PS. After 24 h, the DMEM was removed and the extracts were added at 100%, 50%, 25%, 12.5%, and 6.25% dilution rates. Cell viability was determined using a commercially available XTT kit (Biological Industries, Kibbutz Beit-Haemek, Israel, Catalog #: 20-300-1000) following the kit manufacturer’s instructions. The measurements were made after 24, 48, and 72 h using a Varioskan flash plate reader (Waltham, MA, USA). The percent viability of the cells was calculated in comparison with a control group, where the cells were grown in DMEM with no extracts and 100% viability was assumed.

### 2.4. xCELLigence Real Time Cell Analysis

Real-time cell analysis (RTCA) was performed using the xCELLigence RTCA DP system and 16-well E-plates (Agilent Technologies, Inc., Santa Clara, CA, USA). In total, 100 µL of DMEM containing 10% FBS and 1% PS was added to the plates. After background impedance readings, the cells prepared as described above were at 1 × 10^4^ cells/well with the final volume in each well reaching 200 µL. The cells were allowed to settle at room temperature for 30 min to promote uniform attachment. The measurements were made with no agents added for the first 24 h. At the end of 24 h, the media in the cells were removed and undiluted extracts prepared from the resins were added to the wells in triplicates. The measurements continued for 72 h.

### 2.5. Statistical Analysis

Cell viability data obtained from the XTT assay were analyzed using one-way analysis of variance (ANOVA) to determine significant differences among the study groups. Tukey’s HSD post hoc comparisons were performed to identify pairwise differences. Statistical significance was set at *p* < 0.05. Data analysis was conducted using SPSS v.25. Cell doubling times were calculated using the xCELLigence RTCA software v 2.0.

## 3. Results

**Figure 3:**
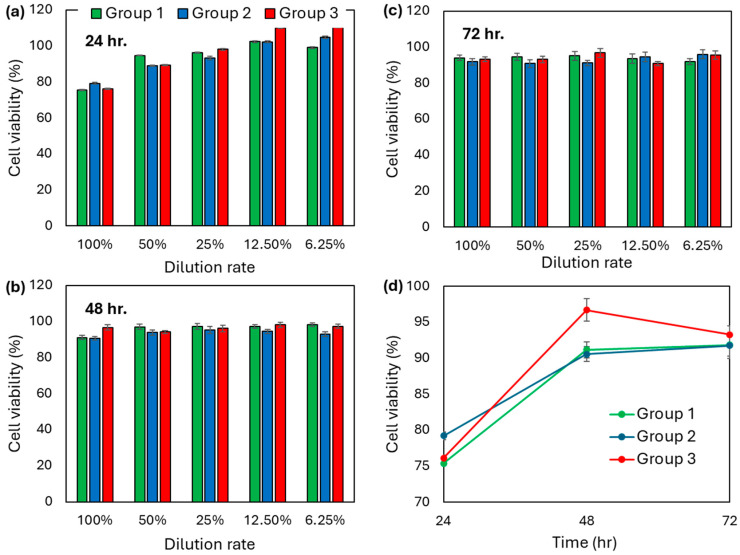
Cell viability of HGF cells exposed to resin extracts relative to the negative control group at (**a**) 24 h, (**b**) 48 h, and (**c**) 72 h obtained from the XTT assay. (**d**) Time-wise cell viability of HGF cells exposed to undiluted extracts. (Group 1: Tera Harz TC-85 DAC resin post-cured using the Graphy THC 2. Group 2: Clear-A resin post-cured in a glycerin-filled glass container using Ackuretta Curie. Group 3: Clear-A resin post-cured using the Graphy THC 2).

The XTT assay results showed that none of the tested samples of the study groups resulted in a cytotoxic effect according to the 70% cell viability limit defined in the ISO 10993-5: Biological Evaluation of Medical Devices—Tests For in vitro Cytotoxicity standard. At 24 h, a 100% dilution rate of the tested samples resulted in a ~75–80% viability in all study groups. A further dilution rate of the tested samples at 24 h showed a cell viability well above 90%. Furthermore, at 48 and 72 h, all dilution rates of the tested samples exhibited no discernible differences in cell viability, in all study groups (Figure 3).

IC_50_ values for Groups 1, 2, and 3 could not be accurately determined, as none of the substances reduced cell viability by 50% within the tested concentration range (6.25–100%) (Table 1). Therefore, the IC_50_ for these compounds was assumed to be >100%, indicating no cytotoxic potential under the conditions of the XTT assay. The highest concentration tested (100%) corresponds to the undiluted form of the extracts, which reflects the maximum physiologically or clinically relevant dose. Therefore, testing concentrations beyond this level was not feasible. To maintain experimental relevance and ensure an accurate interpretation of cytotoxic effects, undiluted (100%) extracts were used for the subsequent xCELLigence RTCA analyses.

**Figure 4:**
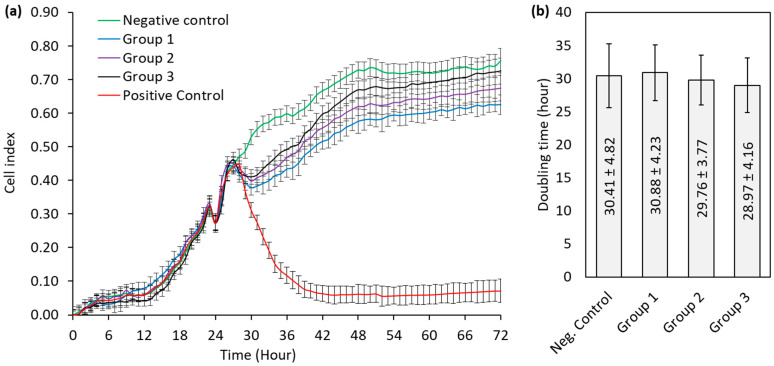
(**a**) Time-dependent cell index plot from the xCELLigence real-time cell analysis (the extracts were added at 24 h). (**b**) Cell doubling times between 30 and 50 h calculated using the xCELLigence real-time cell analysis software. (Group 1: Tera Harz TC-85 DAC resin post-cured using the Graphy THC 2. Group 2: Clear-A resin post-cured in a glass that filled with glycerin using the Ackuretta Curie. Group 3: Clear-A resin post-cured using the Graphy THC 2) (The data labels indicate the average doubling time and the standard deviation of the corresponding groups).

The xCELLigence RTCA results exhibited a slight decline in cell viability during the first 24 h following the addition of the extracts. This was followed by an increase indicating resumed cell proliferation (Figure 4a). The doubling times calculated between 30 and 50 h, during which logarithmic growth was observed after the initial decrease in the cell index, are shown in Figure 4b. All groups exhibited comparable doubling times. The observed differences in doubling times were not statistically significant, indicating no substantial cytotoxic effects from any of the tested materials.

## 4. Discussion

The findings of this study indicate that the extracts of the tested post-processed resins used in the direct production of clear aligners exhibited limited cytotoxicity upon early exposure at high concentrations, followed by cellular recovery under the test conditions. The XTT assay results demonstrated a reduction in cell viability with the undiluted extracts across all groups at 24 h; however, viability remained above the 70% cytotoxicity threshold established by ISO 10993-5. The xCELLigence RTCA results were consistent with the XTT assay results, which also demonstrated a slight decrease in cell viability during the first 24 h after the extracts were added. The agreement between the two independent assays supports the reliability of the findings and further confirms that the initial viability reduction was temporary and did not significantly impact overall cell proliferation. Therefore, the alternative hypothesis was largely rejected.

The in vitro design of this study, incorporating both XTT and real-time xCELLigence RTCA cytotoxicity assays, allowed for a comprehensive evaluation of cellular responses at multiple time points. The inclusion of early (24 h) and later (48 and 72 h) measurements was critical in capturing not only the initial impact of leachates from the post-processed materials, but also the potential for cellular adaptation or recovery over time. This multi-time-point approach is consistent with previous in vitro studies evaluating the short-term cytotoxic effects of 3D-printed dental resins using similar assay combinations [18,19]. Such a design enhances the interpretability of transient cytotoxic effects and provides insight into how short-term exposure might translate to clinical scenarios where aligners come into immediate contact with oral tissues following fabrication.

Both resins used in this study are CE-certified, and TC-85 DAC has additionally received FDA and KFDA approval, supporting their regulatory suitability for clinical applications. However, the observed transient reduction in cell viability under certain post-curing conditions suggests that short-term biological responses may still vary despite compliance with regulatory standards. This observation aligns with earlier findings indicating that regulatory clearance does not always guarantee uniform biological compatibility across different post-processing scenarios [18,19]. Similarly, other studies have shown that residual monomers released from epoxy- or acrylic-based polymers may induce mild cytotoxic effects, which tend to increase with prolonged exposure durations or insufficient post-curing procedures [20,21].

The transient decrease in cell viability observed at 24 h, while still above the cytotoxicity threshold defined by ISO 10993-5, is likely attributable to the initial release of water-soluble leachates such as residual monomers from the 3D-printed materials [4,22]. These compounds are known to elute rapidly upon exposure to aqueous environments, and their presence may temporarily affect cell health in vitro. Similarly, ex vivo studies on thermoformed clear aligners have reported higher levels of bisphenol A release within the first 24 h of exposure [23]. The subsequent increase in cell viability observed at 48 and 72 h suggests a potential cellular adaptation or recovery response. It is possible that cells activate repair mechanisms when exposed to mild, sublethal stressors. From a clinical perspective, this finding suggests that rinsing or soaking clear aligners in water prior to first use may be a simple yet effective approach to reduce initial leachate exposure and improve the material’s biocompatibility for patients.

A potential limitation of extract-based cytotoxicity studies is the possibility that volatile organic compounds, such as unreacted monomers or additives, may evaporate during the extraction process, thereby reducing the concentration of leachates in the test medium. This could result in an underestimation of cytotoxicity, particularly for low-molecular-weight monomers with high volatility. To mitigate this issue, sealed containers were used during the extraction phase to minimize the loss of volatile compounds. Additionally, all test and control groups were incubated under identical conditions to ensure consistent evaporation rates, if any. However, this method does not entirely eliminate the possibility of variation in leachate concentrations. Willi et al. [22] reported that extracts prepared from Tera Harz TC-85 DAC were free of bisphenol A (BPA) but contained quantifiable amounts of urethane dimethacrylate (UDMA), ranging from 29 to 96 µg/L. They noted that the amount of UDMA leached from the samples varied, even under standardized conditions [22]. Future studies could incorporate analytical techniques such as gas chromatography–mass spectrometry (GC-MS) to directly quantify specific monomer concentrations in extracts, providing a more accurate assessment of leachate variability and its potential impact on cytotoxicity.

The biocompatibility of dental materials is a critical consideration, as it determines their capacity to interact with biological systems without inducing adverse oral effects or systemic toxicity [24]. Human-derived cell culture models are widely used to evaluate material biocompatibility due to their ethical acceptability, cost-effectiveness, and scientific relevance [25]. In the present study, HGFs were selected for cytotoxicity testing due to their key role as the predominant cell type within the gingival connective tissue and their involvement in maintaining periodontal tissue homeostasis. They exhibit a wide range of biological functions, including extracellular matrix production, the modulation of immune responses through cytokine secretion, and the facilitation of wound healing [26,27,28]. These characteristics make HGFs a widely accepted in vitro model for evaluating the biocompatibility of dental materials [29].

The XTT assay for cell viability is a well-established method that has been extensively employed to assess the potential cytotoxic effects of substances released from dental materials and devices, including resins used in additive manufacturing for dental applications [4,7,15,19,30]. In contrast, the xCELLigence real-time cell analysis (RTCA) system is a relatively recent approach that enables the continuous and label-free monitoring of cell growth dynamics, although its broader adoption may be constrained by its higher operational costs compared to the XTT assay. Combining both the XTT and xCELLigence RTCA assays offers a robust and complementary strategy for generating reliable preliminary cytotoxicity data, which can serve as a foundation for more comprehensive toxicological evaluations.

Given that 3D-printed clear aligners primarily come into contact with gingival tissues, their cytotoxicity was evaluated. The existing literature includes relatively few studies assessing the cytotoxic effects of 3D-printed clear aligners. To the best of our knowledge, no previous study has assessed their cytotoxicity using a combination of the XTT assay and the xCELLigence real-time cell analysis (RTCA) system. Among the available studies, Iodice et al. [15] incubated cells with resin extracts at dilution rates of 50% and 100% for 72 h, Pratsinis et al. [4] at 20%, 10%, and 5% for 72 h, and Alessandra et al. [7] at 100% for 4 h. In all cases, cell viability was measured at the end of the incubation period. In the present study, we evaluated cytotoxicity at dilution rates of 100%, 50%, 25%, 12.5%, and 6.25% over 24, 48, and 72 h, in accordance with the ISO 10993-5 standard.

The cytotoxicity of 3D-printed materials may be influenced by several factors, including the composition of the resin, the type of printer used, the printing speed, the orientation of the aligner on the build platform, and the washing and post-curing protocols applied [7,11,12,31]. In the present study, all samples were printed using the same 3D printer under identical printing conditions. The cytotoxicity of the Tera Harz TC-85 DAC and Clear-A resins was compared using the same washing and post-curing procedures. Additionally, the impact of different washing and post-curing protocols on cytotoxicity was evaluated using the Clear-A resin.

As far as we are aware, this is the first study to compare the cytotoxicity of the Tera Harz TC-85 DAC and Clear-A resins under standardized invitro conditions. Both resins were subjected to the post-curing protocol recommended for TC-85 DAC. Additionally, Clear-A was processed using a second protocol recommended by its own manufacturer, which involves post-curing in a glycerol-filled glass container. The decision to apply the TC-85 DAC protocol to Clear-A was based on anecdotal user reports suggesting that this method may enhance its material properties. While post-curing protocols have been suggested to affect polymerization efficiency and may reduce residual monomers [32,33], no statistically significant differences in cytotoxicity were observed among the groups in the present study.

The initial decrease in cell viability observed following the addition of the extracts is likely attributable to an early cellular response to components released from the materials. This transient reduction may have been caused by the presence of residual unreacted monomers, curing byproducts, or pH variations in the culture medium, all of which can induce temporary cellular stress. Additionally, oxidative stress and the generation of reactive oxygen species from extractable components may have contributed to the early decline in cell viability. However, the observed recovery and subsequent proliferation of the cells suggest an adaptive response and minimal long-term cytotoxic effects [34,35].

A comprehensive review of the literature identified a limited number of studies investigating the cytotoxicity of 3D-printed clear aligners. Among these, resins such as Tera Harz TC 85 DAC and its comparable variants (e.g., TC-85DAW), fabricated using various 3D printers and post-curing processes, are the primary focus [4,7,14,15,36]. Pratsinis et al. [4] reported no significant differences in cell viability, as assessed by the MTT assay on human gingival fibroblasts, at dilution rates of 20%, 10%, and 5% after 72 h of exposure. In that study, the Sprintray Pro 55 3D printer (Sprintray, Los Angeles, CA, USA) and the Cure M curing device (Graphy, Seoul, Republic of Korea) were used for sample fabrication and post-curing, respectively [4].

In the study by Kim et al. [14], the cytotoxicity of two different cleaning methods was compared using a centrifuge machine at room temperature and at a high temperature for 2, 4, and 6 min, and the viability of L929 cells was evaluated using the MTT assay. An LCD 3D printer (UNIZ NBEE, UNIZ Technology LLC, San Diego, CA, USA) and the THC 2 ultraviolet curing device (Graphy, Seoul, Republic of Korea) were utilized in the study. All groups were found to meet the biocompatibility criteria defined by ISO standards, and an even higher cell viability was observed at 6 min under room-temperature conditions and at 4 min under high-temperature conditions [14]. These findings are consistent with our results for the Tera Harz TC 85 DAC resin [4,14].

The cytotoxicity of aligners produced using two different post-curing devices was evaluated in the study by Alessandra et al. [7], which utilized the AccuFab L4D printer (Shining 3D Tech. Co., Hangzhou, China) and Tera Harz TC 85 DAC resin. One group was post-cured for 14 min using the THC device, while the other group was post-cured for 30 min using the Form Cure unit (FormLabs Inc., Somerville, MA, USA). The viability of MC3T3-E1 preosteoblast cells was assessed on days 7 and 14 using the MTT assay. At both time points, a significantly lower cell viability was observed in the Tera Harz Cure group compared to the Form Cure group [7].

In our study, we observed no cytotoxic effect at both 10-min post-curing with the Curie machine and 25-min post-curing with the THC2for Clear-A resin. The differences between our results and those of Alessandra et al. [7] might result from the type of printer and resin used and the cells evaluated. In our opinion, the reason might not be the different post-curing durations. Iodice et al.’s study [15] examined the cytotoxicity of three post-curing durations (14, 24, and 50 min) for the Tera Harz Cure device, and the results indicated that while the post-curing durations of 14 and 24 min did not differ significantly, 50-min post-curing significantly decreased cell viability [15].

While this study followed the ISO 10993-5 guidelines for cytotoxicity testing, including the recommended extraction ratio of 6 cm^2^/mL, it is important to note that the sample dimensions used do not represent the full surface area of clinically applied upper and lower aligners. Consequently, the total amount of leachates released in vivo may differ from those observed under these standardized in vitro conditions. Therefore, the cytotoxicity findings presented here should be interpreted as indicative of the material’s relative biocompatibility under controlled testing conditions, rather than as a direct prediction of clinical safety.

To the best of our knowledge, this is the first study to assess the cytotoxicity of Clear-A resin using two distinct post-processing protocols. Although TC-85 DAC resin is generally considered biocompatible when printed and post-cured according to the manufacturer’s instructions, oral mucosa irritation has been reported in some patients, and at least one case of a severe allergic reaction has been documented during clinical use [15,37].

Although a wide variety of resins are available for dental applications, the number of resins specifically designed for the fabrication of clear aligners remains limited, despite recent increases [37,38]. Previous studies have often focused on resins intended for other purposes, such as splint fabrication, which complicates direct comparisons with the present study [29,39,40]. Additionally, some of these studies employed isopropyl alcohol (IPA) to remove uncured resin residues after printing [29,39].

However, the use of IPA is not recommended for resins used in clear aligner fabrication, including those examined in this study, due to concerns that it may compromise the mechanical properties of the printed material. A resin-specific approach to post-processing is also noteworthy. Similarly, for TC-85 DAC, rinsing is recommended using a water-based centrifugation process, followed by post-curing in a nitrogen-filled chamber with a specialized curing device. For Clear-A resin, rinsing with water through centrifugation is advised, followed by post-curing in a glycerin-filled container.

The use of 70% ethanol for disinfection may have had a minor influence on the surface properties of the 3D-printed samples. However, this method was intentionally selected to avoid confounding factors such as residual ethylene oxide by-products or UV-induced alterations, which could independently affect cytotoxicity. Moreover, ethanol or isopropanol immersion is consistent with established post-processing protocols [14,41,42].

In this study, all samples were fabricated by an experienced operator with approximately five years of expertise in 3D printing workflows. To ensure consistency and reproducibility, only resins with which the operator was thoroughly familiar, particularly in terms of printing and post-processing procedures, were included. Resins that required handling protocols unfamiliar to the team were deliberately excluded.

In this regard, only two types of clinically approved resins were evaluated, and no chemical characterization of the resin extracts was conducted to identify specific cytotoxic components. Future studies are encouraged to include a broader range of resins specifically developed for direct aligner fabrication and to employ advanced analytical techniques such as chromatography and mass spectrometry to better elucidate the potential causes of cytotoxicity.

Additionally, exploring alternative sterilization or disinfection methods may provide further insight into the material behavior and surface biocompatibility of these resins. Investigations using full-size aligners and dynamic intraoral simulation models are also strongly recommended to better assess the long-term biological effects of aligner materials under clinically relevant conditions.

Another limitation of the present study is the short-term evaluation using only human gingival fibroblast (HGF) cells. While in vitro models based on HGF cells and human saliva are designed to replicate aspects of the intraoral environment, they fail to account for critical factors such as mechanical loading during mastication or bruxism, dietary chemical exposure, and thermal fluctuations. Furthermore, reliance on a single cell type may not adequately reflect the diverse cellular responses present within the oral cavity.

Therefore, future studies should consider incorporating additional relevant oral cell lines, including oral keratinocytes and periodontal ligament fibroblasts, and extending incubation durations to assess potential long-term or cumulative effects. Together, these strategies will contribute to a more comprehensive understanding of the biological safety of aligner materials and their associated post-processing protocols.

## 5. Conclusions

1. The XTT assay results demonstrated that the cell viability of all study groups remained above the 70% cytotoxicity threshold defined by ISO 10993-5 across all dilution rates and time points. However, a slight decrease in cell viability was observed at 24 h in the undiluted extract group.
2. The xCELLigence RTCA analysis similarly revealed a temporary reduction in cell viability within the first 24 h, which was subsequently followed by continuous cell proliferation over time.
3. These findings indicate that the tested resins and post-curing protocols exhibited no cytotoxic effects on human gingival fibroblasts, supporting their suitability for continued development in dental and biomedical applications.
4. The evaluation of resins used in 3D clear aligner production with both assays resulted in consistent findings that provide preliminary evidence for the biological safety of these materials and suggest their potential suitability for clinical use.

## Figures and Tables

**Table 1:** IC_50_ values (%) of three test groups at 24, 48, and 72 h based on XTT assay results.

Group	Time	IC_50_ (%)
**Group 1**	24 h	185.98
	48 h	227.61
	72 h	221.25
**Group 2**	24 h	186.95
	48 h	221.98
	72 h	219.35
**Group 3**	24 h	202.16
	48 h	196.90
	72 h	223.75

**Group 1**: Tera Harz TC-85 DAC resin post-cured using the Graphy THC 2. **Group 2**: Clear-A resin post-cured in a glycerin-filled glass container using Ackuretta Curie. **Group 3**: Clear-A resin post-cured using Graphy THC 2.

## Data Availability

The data that support the findings of this study are available from the corresponding author upon reasonable request.
